# Enhancing Effect of Borneol and Muscone on Geniposide Transport across the Human Nasal Epithelial Cell Monolayer

**DOI:** 10.1371/journal.pone.0101414

**Published:** 2014-07-03

**Authors:** Zhenzhen Chen, Xin Gong, Yang Lu, Shouying Du, Zhihui Yang, Jie Bai, Pengyue Li, Huichao Wu

**Affiliations:** 1 School of Chinese Materia Medica, Beijing University of Chinese Medicine, Beijing, China; 2 Reproductive Endocrinology Centre, Dongfang Hospital of Beijing University of Chinese Medicine, Beijing, China; 3 Department of Psychiatry, University of Florida, Gainesville, Florida, United States of America; Université Pierre et Marie Curie-Paris6, INSERM, CNRS, France

## Abstract

Geniposide is widely used in the treatment of cerebral ischemic stroke and cerebrovascular diseases for its anti-thrombotic and anti-inflammatory effects. Recent studies demonstrated that geniposide could be absorbed promptly and thoroughly by intranasal administration in mice and basically transported into the brain. Here, we explored its transport mechanism and the effect of borneol and muscone on its transport by human nasal epithelial cell (HNEC) monolayer. The cytotoxicity of geniposide, borneol, muscone and their combinations on HNECs was evaluated by the MTT assay. Transcellular transport of geniposide and the influence of borneol and muscone were studied using the HNEC monolayer. Immunostaining and transepithelial electrical resistance were measured to assess the integrity of the monolayer. The membrane fluidity of HNEC was evaluated by fluorescence recovery after photobleaching. Geniposide showed relatively poor absorption in the HNEC monolayer and it was not a P-gp substrate. Geniposide transport in both directions significantly increased when co-administrated with increasing concentrations of borneol and muscone. The enhancing effect of borneol and muscone on geniposide transport across the HNEC may be attributed to the significant enhancement on cell membrane fluidity, disassembly effect on tight junction integrity and the process was reversible. These results indicated that intranasal administration has good potential to treat cerebrovascular diseases.

## Introduction

Geniposide, an iridoid glycoside isolated from Gardenia, is one of the main active ingredients in “*Xing Nao Jing*”, an herbal injection extracted from a traditional Chinese herbal medicine recipe “*An Gong Niu Huang Wan*” that has been used clinically in stroke treatment for hundreds of years. However, delivering geniposide to the brain is a significant challenge due to the presence of both the blood-brain and the blood-cerebrospinal fluid barriers. According to the results of a preliminary study [1], the absorption percentage of geniposide in the intestinal tract of rats was only about 15.9% at 3 h, and the apparent partition coefficient of geniposide between octanol and water was about 0.108. Our previous studies have shown that the brain/blood drug ratio of geniposide via intravenous administration in mice was only about 10% [2]. Therefore, there is an urgent need to develop a simple, patient compliant and effective method of delivering geniposide to the brain for the treatment of brain disease.

Intranasal administration has been studied as a potential strategy for enhancing the delivery of drugs to the brain [3], [4]. This route of administration is relatively more patient compliant and noninvasive than injection, thus allowing for more frequent administration. The nasal mucosa has a relatively large surface area due to the numerous microvilli, relatively high apparent permeability to both hydrophilic and lipophilic compounds (at least for those smaller than 1000 Da), and is covered by a thin epithelium [5], [6]. Drugs are directly targeted to the Central Nervous System (CNS) with intranasal delivery, reducing systemic exposure and thus unwanted systemic side effects. Delivery from the nose to the CNS occurs within minutes along both the olfactory [7], and trigeminal [9], [10] neural pathways via an extracellular route and does not require drug to bind to any receptor or axonal transport. Since drugs could be directly delivered into CNS bypassing the blood-brain barrier via intranasal administration, this route was considered to be an attractive alternative to traditional injection therapy for CNS disorders. Previous studies have shown that borneol could enhance drug permeation through skin [11], gastrointestinal mucous membrane [12], nasal mucosa and cornea [13], [14].

Introducing a new substance for nasal administration requires biopharmaceutical studies such as bioavailability studies. Before in vivo preclinical testing, it is reasonable to screen possible drug candidates with respect to their permeability through the nasal mucosa using an appropriate in vitro model. To study drug transport and permeation through the nasal mucosa, different in vitro nasal mucosa models have been developed [15]–[17]. Although in vitro and in vivo animal models have been widely used for nasal delivery studies, they show a significantly different nasal cavity structure compared to humans, which sometimes misleads in predicting drug uptake and absorption [18]. In vitro nasal cell culture model has attracted the attention of pharmaceutical researchers as a promising tool for defining transport mechanisms and testing novel strategies to enhance drug transport and absorption. Serially passaged human nasal epithelial cell monolayer has made high-throughput screening studies possible [19].

The purpose of the present work was to characterize the transport mechanism of geniposide using human nasal epithelial cells in primary culture and to evaluate the influence of borneol and muscone on its transport through this membrane model. Geniposide showed relatively poor absorption in the HNEC monolayer and was not a P-gp substrate. Geniposide transport in both directions significantly increased when co-administrated with increasing concentrations of borneol and muscone.

## Materials and Methods

### 2.1. Ethics statement

All experimental protocols were approved by the Ethics Committee of the Dongfang Hospital Human Ethics Committee (NO. 2011090205). Written informed consent was obtained from all participants of this study.

### 2.2. Materials

Polyester (PET) transwell plates (12 mm diameter, 0.4 µm pore size) were purchased from Corning Costar Corporation (MA, USA). Geniposide was obtained from the National Institute for the Control of Pharmaceutical and Biological Products (Beijing, China). Borneol was purchased from Guizhou Golden Pharmaceutical Co. Ltd. Muscone was obtained from the National Institute for the Control of Pharmaceutical and Biological Products (Beijing, China). Acetonitrile (Mreda Inc, USA) was of HPLC grade. Acti-stain 488 Fluorescent phalloidin was purchased from Cytoskeleton Corp. Bronchial epithelial cell growth medium (BEGM) bullet kit was obtained from Clonetics Corp.

### 2.3. Isolation and cultivation of HNECs

A reported method on the cultured HNECs was slightly modified [19]. Briefly, the tissues were treated with 1.0% protease (type XIV, Sigma) supplemented with 100 U·mL^−1^ penicillin and 100 µg·mL^−1^ streptomycin for 1 h at 37 °C. Tissue debris was removed by passing the cell suspension through a 40 µm cell strainer. Dissociated epithelial cells were washed three times with DMEM/F12 containing antibiotics and then suspended in the same medium supplemented with 10% fetal bovine serum. Cells were pre-plated on a plastic dish at 37 °C for 1 h in order to eliminate the majority of fibroblasts, endothelial cells and myoblasts by differential attachment to the bottom of the plastic well. Suspended epithelial cells were cultured in BEGM at 37 °C in an atmosphere of 5% CO_2_ and 95% relative humidity. The medium was changed every 2 days until cells reached about 90% confluence.

### 2.4. Isolation of HNECs from a mixed population of cells

HNECs were positively selected from the above heterogeneous cell population using a Dynabeads epithelial enrich (Invitrogen). In brief, cells were suspended in HBSS containing 1% BSA and 2 mM EDTA. Volume of the suspension medium was adjusted so that the cell concentration was 2×10^7^. Magnetic nanoparticles at 50 µL·mL^−1^ amount were added to the cell suspension and incubated for 30 min at 4 °C. HNECs were separated by placing the tube in a magnetic block for 2 min. HNECs attached to the walls of the tube and were separated by discarding the cell suspension. Lastly, the bead free HNECs were obtained using reconstituted release buffer (DNase I) and were subsequently seeded in a 25 cm^2^ T flask with renewed medium every 2 days. Subcultures were established through trypsin treatment and reseeding. Cells were passaged in a 1∶3 split ratio. The passage 5–8 HNECs were used in the following experiments.

### 2.5. Cytotoxicity assays

The cytotoxicity of the main active ingredients in “*Xing Nao Jing*” on HNECs was evaluated by the methyl thiazolyl tetrazolium (MTT) colorimetric assay [20]. Briefly, 100 µL of 5×10^4^ cells·mL^−1^ was seeded into 96-well plates. After 24 h, the medium was removed and replaced with a fresh medium containing increasing concentrations of geniposide, borneol, muscone and their combinations for another 24 h. After that, 15 µL of a 5 mg·mL^−1^ MTT solution in 100 µL BEGM was added to each well. After 4 h of incubation, the supernatant was discarded and formazan crystals were dissolved in DMSO followed by vigorous mixing. Control wells were incubated with BEGM only and were treated similarly as above. The optical density was determined at 490 nm with a Multiskan Go microplate reader (Thermo, USA). The percent viability of the cells was determined from the absorbance values considering that of the control as 100%.

### 2.6. Transport studies of geniposide

When HNECs reached approximately 80–90% confluence, the cells were detached with trypsin and were seeded at a density of 2×10^5^ cells·cm^−2^ on PET inserts. The culture medium was BEGM:DMEM/F12 (1∶1) in both sides at 37 °C in an atmosphere of 5% CO_2_ and 95% relative humidity, which was first changed after 24 h of seeding and then every 2 days.

The transport experiments were carried out when transepithelial electrical resistance (TEER) value was higher than 500 Ω·cm^2^. The transport studies were performed by initially incubating the monolayers in Hanks balanced salt solution (HBSS) for 30 min at 37 °C. To measure apical (A) → basolateral (B) transport, 0.5 mL of drug solution and 1.5 mL of HBSS were added in the A and B sides, respectively. Basolateral (B) → apical (A) transport was evaluated by adding 1.5 mL of drug solution in B side and 0.5 mL of HBSS in A side [21]. The final organic solvent concentration in HBSS was always kept below 1%, a concentration which did not alter cell viability or permeability [22].

Geniposide transport was studied at three concentrations: 25, 50 and 100 µg·mL^−1^. The effect of borneol was studied at 27.8, 55.6 and 111.2 µg·mL^−1^ concentrations in the presence of 50 µg·mL^−1^ geniposide. The effect of muscone was studied at 4.17, 8.34 and 16.68 µg·mL^−1^ concentrations in the presence of 50 µg·mL^−1^ geniposide. Finally, the effects of both borneol and muscone were studied at the concentrations mentioned above in the presence of 50 µg·mL^−1^ geniposide.

Each measurement was evaluated in triplicate. Cells were incubated in a 37 °C shaking incubator. Samples of 200 µL were taken from the apical side (to study B → A transport) and samples of 600 µL were taken from the basal side (to study A → B transport) at 30, 60, 90, 120, 150, 180 min time intervals. The same volume of fresh preheated HBSS was added to keep the volume constant. Samples were centrifuged for 10 min at 12,000 r·min^−1^ and the supernatant was analyzed for geniposide by HPLC.

### 2.7. HPLC measurement of geniposide

An UV-HPLC method was used to quantify the geniposide concentrations in the transport studies. The chromatographic conditions consisted of a Purospher Star C18 column (4×200 mm, particle size 5 µm) plus guard column. Geniposide samples were analyzed with UV detection (*λ* = 238 nm) and the mobile phase was composed of acetonitrile and water (16∶84, v/v). The mobile phase was pumped at a flow rate of 1 mL·min^−1^ and the injected volume was 20 µL. In these conditions, the retention time of geniposide was 8.5 min.

### 2.8. Immunostaining

Cells cultured on 12-well polyester membrane Transwell clear inserts were stained for actin. Seven days later, for immunolabeling, the cells were fixed for 20 min in 4% paraformaldehyde/phosphate buffered saline (PBS), permeabilized with 1% Triton X-100 for 10 min, stained with Acti-stain 488 Fluorescent phalloidin for 30 min, washed three times with PBS, and mounted in buffered glycerol. Fluorescence was visualized on an epifluorescence microscope (IX-71, Olympus, Japan) using a blue filter and images were taken using the analysis software.

### 2.9. TEER measurement

Transepithelial electrical resistance was measured using an EVOM instrument (Millipore Corporation, USA) to check the monolayer integrity. The TEER of untreated cells and cells treated with borneol and muscone was determined in BEGM:DMEM/F12 (1∶1) before the experiment, after the experiment and after 24 h. The measured TEER before the experiment was set as 100% and all other values were calculated according to this.

### 2.10. Visualizations of membrane fluidity of HNECs

The cells were seeded on cell plate at a density of 5×10^4^ cells·cm^−2^. The above formulation groups were added and the plates were incubated for 3 h at 37 °C in a 5% CO_2_ incubator. Cells were rinsed with HBSS and stained with NBD-C_6_-HPC at 2 µg·mL^−1^ for 30 min.

A method of fluorescence recovery after photobleaching (FRAP) was used here. After the definition of line scanning position, length and photobleaching position, the monolayer was visualized by a Confocal Laser Scanning Microscope under the following conditions: excitation wavelength of 488 nm, emission wavelength of 530 nm, laser power for fluorescence bleaching of 100%, bleaching time of 0.7 s, scanning frequency of 1.65 s and the total scanning time of 100 s. The fluorescence intensity time-variation at the photobleaching position was recorded and image formation was obtained by confocal tomoscanning.

### 2.11. Statistical analyses

The percentage of cell toxicity in the MTT assay was calculated by the following equation (1):
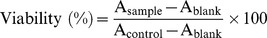



The apparent permeability coefficients (*P_app_*) for geniposide were calculated according to the following equation (2):
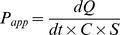
where *P_app_* is the permeability, *dQ/dt* is the apparent appearance rate of drug in the receiver side calculated using linear regression of amounts in the receiver chamber versus time, *C* is the drug concentration in the donor chamber and *S* is the surface area of the monolayer. The efflux ratio (*ER*) was calculated according to the following equation (3):
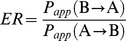



The membrane fluidity was represented by fluorescence recovery rate (*R*) which was calculated with the following equation (4): 
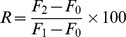
where *F_0_* is the instant fluorescence value after photobleaching, *F_1_* is the fluorescence value before photobleaching and *F_2_* is the fluorescence recovery value after photobleaching.

Data were expressed as mean ± SD. Statistical evaluation was carried out by one-way analysis of variance (ANOVA, followed by LSD test) (SPSS17.0 Statistical software). Significance was set at *P*<0.05.

## Results

### 3.1. Cytotoxicity studies

**Figure 1:**
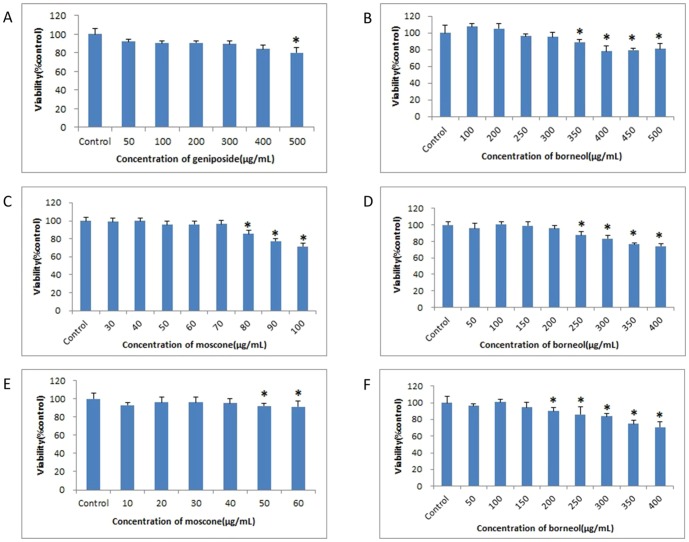
Cytotoxicity of geniposide, borneol, muscone and their combinations determined by MTT test. (A) Geniposide has no cell cytotoxicity in the concentration range of 0–400 µg·mL^−1^. (B) Borneol has no cell cytotoxicity in the concentration range of 0–300 µg·mL^−1^. (C) Muscone has no cell cytotoxicity in the concentration range of 0–70 µg·mL^−1^. (D) GB group (geniposide:borneol, 0.9∶1, w/w) has no cell cytotoxicity in the concentration range of 0–200 µg·mL^−1^ calculated by borneol. (E) GM group (geniposide:muscone, 6∶1, w/w) has no cell cytotoxicity in the concentration range of 0–40 µg·mL^−1^ calculated by muscone. (F) GBM group (geniposide:borneol:muscone, 0.9∶1∶0.15, w/w/w) has no cell cytotoxicity in the concentration range of 0–150 µg·mL^−1^ calculated by borneol. Date are expressed as mean ± SD (*n* = 5). * *P*<0.05 compared with the control group.

Results obtained for HNECs were showed in Figure 1. Geniposide, borneol and muscone groups have no cell cytotoxicity in the concentration range of 0–400, 0–300 and 0–70 µg·mL^−1^ respectively. GB group (geniposide:borneol, 0.9∶1, w/w) has no cell cytotoxicity in the concentration range of 0–200 µg·mL^−1^ calculated by borneol. GM group (geniposide:muscone, 6∶1, w/w) has no cell cytotoxicity in the concentration range of 0–40 µg·mL^−1^ calculated by muscone. GBM group (geniposide:borneol:muscone, 0.9∶1∶0.15, w/w/w) has no cell cytotoxicity in the concentration range of 0–150 µg·mL^−1^ calculated by borneol.

### 3.2. Effect of borneol and muscone on transepithelial permeability of geniposide

Permeability assays for geniposide at concentrations of 25, 50 and 100 µg·mL^−1^ were performed in the HNEC monolayer. To determine whether geniposide transport was polarized, transepithelial fluxes were measured in both directions. As shown in Table 1, geniposide transport does not change significantly in both directions (B→A and A→B) in the HNEC monolayer. The (B→A)/(A→B) permeability *ER* of geniposide was less than 2 in the cell model.

**Table 1:** Various concentrations of geniposide permeability across the HNEC monolayer.

Condition	*P_app_*±SD(A→B)(×10^−6^ cm·s^−1^)	*P_app_*±SD(B→A)(×10^−6^ cm·s^−1^)	*ER*±SD(B→A/A→B)
25 µg·mL^−1^	1.267±0.314	1.346±0.113	1.104±0.256
50 µg·mL^−1^	1.221±0.015	1.260±0.084	1.032±0.076
100 µg·mL^−1^	1.255±0.074	1.188±0.104	0.951±0.132

*P*_app_, apparent permeability; A, apical side; B, basolateral side.

Values are mean ± SD (*n* = 3).

**Figure 2:**
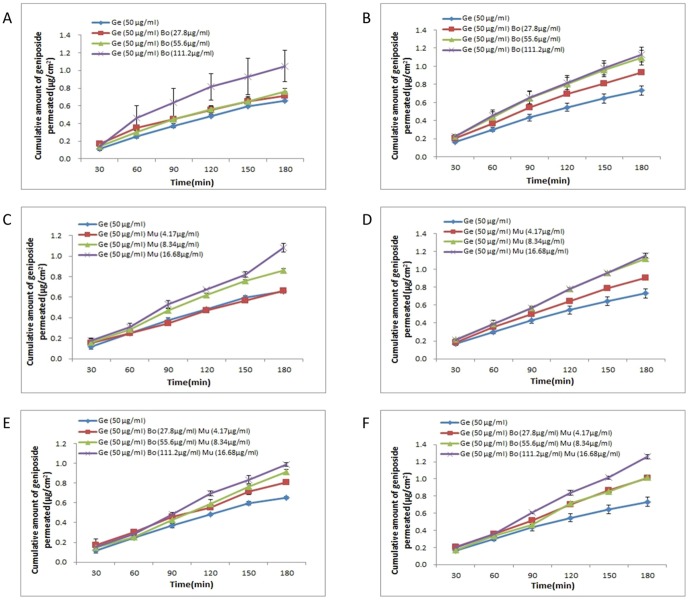
Effects of borneol (Bo) and muscone (Mu) on geniposide (Ge) transport across the HNEC monolayer. (A) Geniposide (50 µg·mL^−1^) combined with borneol A to B transport. (B) Geniposide (50 µg·mL^−1^) combined with borneol B to A transport. (C) Geniposide (50 µg·mL^−1^) combined with muscone A to B transport. (D) Geniposide (50 µg·mL^−1^) combined with muscone B to A transport. (E) Geniposide (50 µg·mL^−1^) combined with borneol and muscone A to B transport. (F) Geniposide (50 µg·mL^−1^) combined with borneol and muscone B to A transport. Date are expressed as mean ± SD (*n* = 3).

The *P_app_* values of geniposide increased with rising concentrations of borneol and muscone in a dose-dependent manner, and were significantly higher than the control (geniposide 50 µg·mL^−1^) (Figure 2). In presence of borneol (111.2 µg·mL^−1^),the A→B fluxes of geniposide in the HNEC were significantly increased (*P*<0.05). When combined with borneol (27.8, 55.6, 111.2 µg·mL^−1^), the B→A fluxes of geniposide in the HNEC were significantly increased (*P*<0.05). In presence of muscone (8.34, 16.68 µg·mL^−1^),the A→B fluxes of geniposide in the HNEC were significantly increased (*P*<0.05). When combined with muscone (4.17, 8.34, 16.68 µg·mL^−1^), the B→A fluxes of geniposide in the HNEC were significantly increased (*P*<0.05). When geniposide transport experiments in the HNEC monolayer were performed in the presence of borneol and muscone, an increasing of permeability values in both directions was observed in a concentration-dependent manner (Bo 27.8 µg·mL^−1^ and Mu 4.17 µg·mL^−1^, *P*<0.05; Bo 55.6 µg·mL^−1^ and Mu 8.34 µg·mL^−1^, *P*<0.05; Bo 111.2 µg·mL^−1^ and Mu 16.68 µg·mL^−1^, *P*<0.05) (Table 2).

**Table 2:** Effects of borneol (Bo) and muscone (Mu) on geniposide (Ge) transport in the HNEC monolayer.

Condition	*P_app_*±SD(A→B)(×10^−6^ cm·s^−1^)	*P_app_*±SD(B→A)(×10^−6^ cm·s^−1^)	*ER*±SD(B→A/A→B)
Ge (50 µg·mL^−1^) (control)	1.221±0.015	1.260±0.084	1.032±0.076
+Bo (27.8 µg·mL^−1^)	1.172±0.120	1.614±0.034*	1.388±0.162
+Bo (55.6 µg·mL^−1^)	1.346±0.086	1.917±0.111*	1.429±0.149
+Bo (111.2 µg·mL^−1^)	1.987±0.293*	1.992±0.126*	1.012±0.106
+Mu (4.17 µg·mL^−1^)	1.147±0.068	1.603±0.021*	1.400±0.071
+Mu (8.34 µg·mL^−1^)	1.609±0.054*	2.039±0.018*	1.268±0.033
+Mu (16.68 µg·mL^−1^)	1.967±0.108*	2.104±0.020*	1.072±0.069
+Bo (27.8 µg·mL^−1^) Mu (4.17 µg·mL^−1^)	1.434±0.083*	1.815±0.024*	1.268±0.057
+Bo (55.6 µg·mL^−1^) Mu (8.34 µg·mL^−1^)	1.755±0.043*	1.916±0.019*	1.092±0.023
+Bo (111.2 µg·mL^−1^) Mu (16.68 µg·mL^−1^)	1.918±0.049*	2.386±0.052*	1.245±0.026

*P*_app_, apparent permeability; A, apical side; B, basolateral side.

Values are mean ± SD (*n* = 3). Differs from Ge (50 µg·mL^−1^), * *P*<0.05.

### 3.3. Effect of borneol and muscone on the actin staining

**Figure 3:**
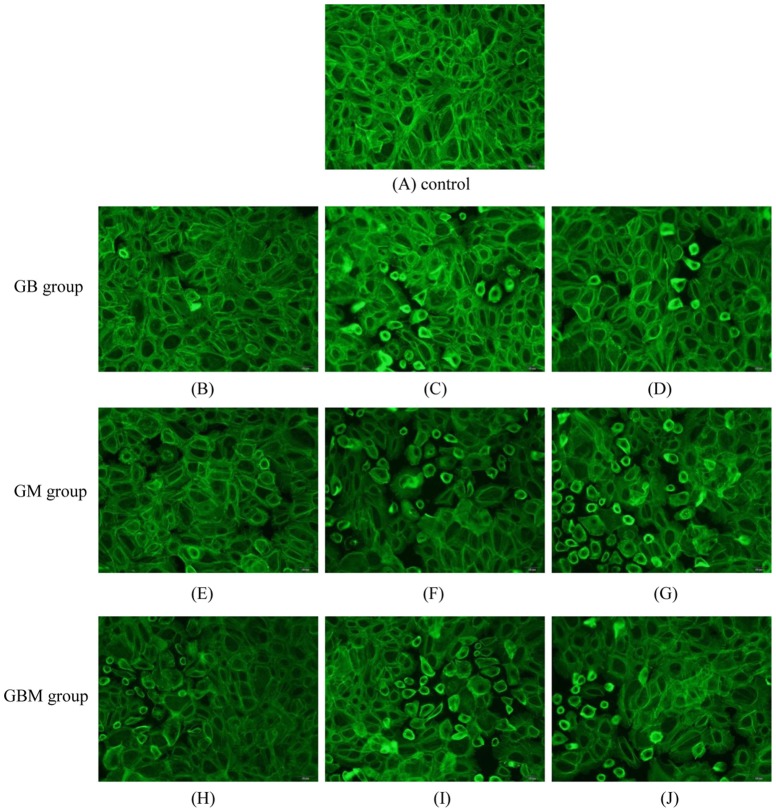
Actin distribution in HNEC monolayer treated with borneol (Bo) and muscone (Mu). (A) 50 µg·mL^−1^ geniposide (control). (B) (C) and (D) 50 µg·mL^−1^ geniposide combined with 27.8, 55.6 and 111.2 µg·mL^−1^ borneol, respectively. (E) (F) and (G) 50 µg·mL^−1^ geniposide combined with 4.17, 8.34 and 16.68 µg·mL^−1^ muscone, respectively. (H) (I) and (J) 50 µg·mL^−1^ geniposide combined with borneol and muscone (Bo 27.8 and Mu 4.17 µg·mL^−1^, Bo 55.6 and Mu 8.34 µg·mL^−1^, Bo 111.2 and Mu 16.68 µg·mL^−1^, respectively). Scale bar: 20 µm.

The distribution of TJ proteins was visualized by immunostaining 3 h after monolayers exposed to geniposide (50 µg·mL^−1^) combined with various concentrations of borneol and muscone (Figure 3). Positive staining for the integral membrane TJ proteins' actin was demonstrated in the HNEC. In the control group, actin formed a circumferential ring parallel to the cell membrane and the contour line appeared lighter and thicker than other groups. The levels of the TJ proteins localized in the cell-cell junctions were decreased in cells exposed to borneol or muscone. In particular, the intensities of actin were largely reduced after cells were exposed to borneol and muscone. Moreover, the GBM group showed fragmented staining for actin, suggesting an impairment of the intercellular junctions.

### 3.4. Effect of borneol and muscone on TEER value

**Figure 4:**
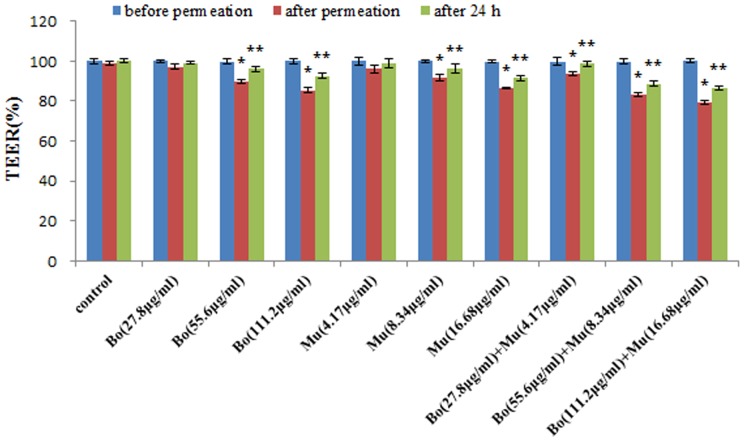
TEER of HNEC monolayer before permeation, after permeation, and after 24 h. Date are expressed as mean ± SD (*n* = 3). * *P*<0.05, TEER of after permeation compared with before permeation, ** *P*<0.05, TEER of after 24 h compared with after permeation.

The integrity of the HNEC monolayer was determined at the beginning, at the end of the experiment and after 24 h. The average TEER values of previous permeation experiments was 528 Ω·cm^2^, which is consistent with the literature reported [23], indicating that the HNEC cell monolayer could be useful for in vitro transport studies. Comparison of the TEER of the cell monolayers in the absence or presence of borneol and muscone were shown in Figure 4. Before the experiment the TEER of all samples was the same and TEER in wells with cells without borneol and muscone did not change during and after the experiment. TEER of cells treated with borneol (55.6, 111.2 µg·mL^−1^), muscone (8.34, 16.68 µg·mL^−1^) and their combinations decreased significantly (*P*<0.05) by 10–20% of the initial value during permeation studies. After washing and incubating the cells with BEGM:DMEM/F12 (1∶1) for 24 h, TEER increased again.

### 3.5. Effect of borneol and muscone on the membrane fluidity of HNECs

**Figure 5:**
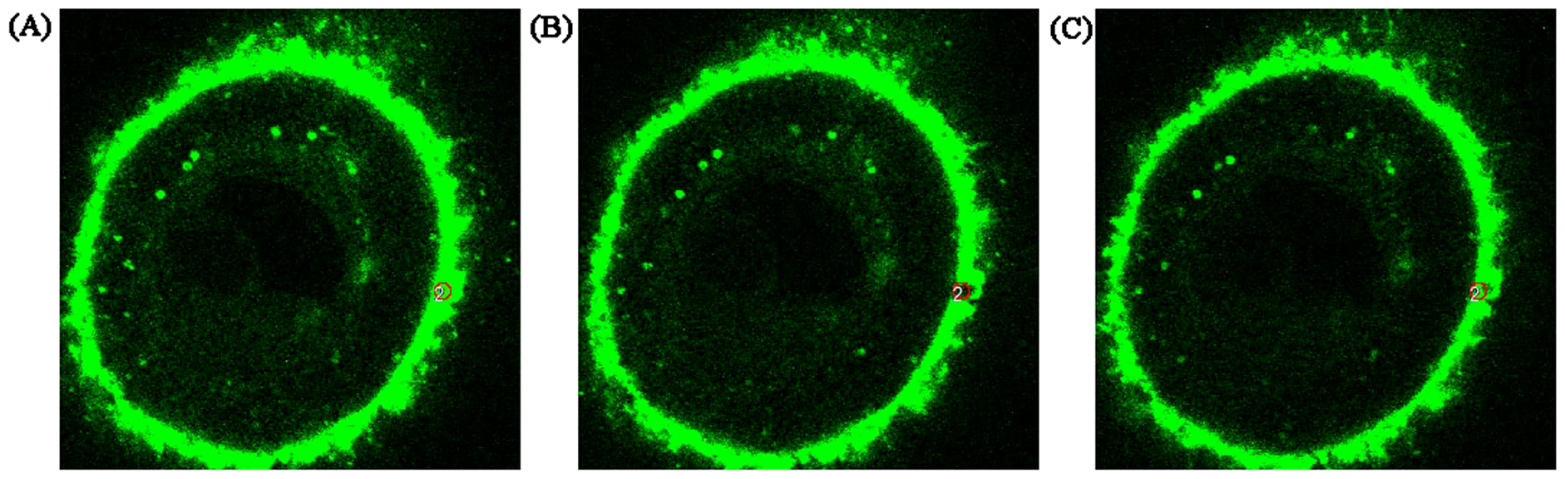
Fluorescence images of membrane phospholipids of HNECs by tomoscan. (A) Before photobleaching, the fluorescence on membrane phospholipids of HNEC was green and well-distributed. (B) After photobleaching, the fluorescence disappeared. (C) Due to membrane fluidity, the fluorescence probe from other areas can move to the photobleaching position leading to the recovery of fluorescence.

Figure 5A shows that the fluorescence on membrane phospholipids of HNEC was green and well distributed. After photobleaching, the fluorescence disappeared (Figure 5B) and the fluorescence recovery happened afterwards at the chosen area (Figure 5C). The fluorescence recovery rate (*R*%) of each group is listed in Table 3. Compared with the control, *R*% of geniposide (50 µg·mL^−1^) combined with borneol (55.6, 111.2 µg·mL^−1^), muscone (8.34, 16.68 µg·mL^−1^) and their combinations had been increased at considerable extent, suggesting their significant enhancement on cell membrane fluidity (*P*<0.05).

**Table 3:** Fluorescence recovery rate after treatment with borneol (Bo) and muscone (Mu).

Condition	*R* (%)±SD
Ge (50 µg·mL^−1^) (control)	33.08±1.74
+Bo (27.8 µg·mL^−1^)	37.08±5.05
+Bo (55.6 µg·mL^−1^)	49.60±4.21*
+Bo (111.2 µg·mL^−1^)	57.33±4.62*
+Mu (4.17 µg·mL^−1^)	36.43±3.37
+Mu (8.34 µg·mL^−1^)	45.45±1.68*
+Mu (16.68 µg·mL^−1^)	49.77±5.63*
+Bo (27.8 µg·mL^−1^) Mu (4.17 µg·mL^−1^)	42.55±0.53*
+Bo (55.6 µg·mL^−1^) Mu (8.34 µg·mL^−1^)	52.34±0.32*
+Bo (111.2 µg·mL^−1^) Mu (16.68 µg·mL^−1^)	66.10±3.78*

Values are mean ± SD (*n* = 3). Differs from Ge (50 µg·mL^−1^), * *P*<0.05.

## Discussion and Conclusions

The intranasal pathway has been proposed as a non-invasive alternative route to deliver therapeutics to the brain. This route will bypass the blood-brain barrier and limit systemic side effects. Upon presentation at the nasal cavity, pharmacological agents reach the brain via the olfactory and trigeminal nerves [24]. Since the nasal epithelium plays an important role in defense against mucosal infections [25], [26], it is particularly important to evaluate the toxicity of compounds in nasal epithelial cells when studying nasal drug formulations. The cytotoxicity of geniposide, borneol, muscone and their combinations were evaluated in the HNECs with the aid of the MTT assay, which consisted of the colorimetric determination of cell viability during treatment with a drug [27]. Results from cytotoxicity assays showed that the cytotoxicity of geniposide was less than that of borneol and muscone at the same concentration. The cytotoxicity of their combination was stronger than their individual cytotoxicity in a concentration-dependent manner. Concentrations that showed no cell cytotoxicity were used in all of the following experiments.

Primary cultured cells have been used for in vitro nasal drug transport studies because first passage cells are believed to best emulate actual human nasal cells in their morphology, phenotype and integrity. Geniposide is a water soluble compound with low molecular weight (388.36). It is hard for geniposide to permeate the HNEC monolayer by a transcellular pathway, but it can across the membrane through the intercellular passage. Geniposide showed relatively poor absorption in the HNEC monolayer, with permeability coefficients ranging from 1.221×10^−6^ to 1.267×10^−6^ cm·s^−1^. The *ER* of geniposide was less than 2 in the cell model. These results suggested that geniposide transport in both directions across the HNEC monolayer was not concentration-dependent and saturable, indicating purely passive diffusion. The *P_app_* values of geniposide in this study were in agreement with previously published data, reporting 0.92 (±0.44)×10^−6^ cm·s^−1^ in primary cultured human bronchial epithelial cell monolayer of the hydrophilic compound [28]. The permeability coefficients may vary among types of cell lines due to differing physiological properties, including the formation of tight junctions.

Borneol and muscone are traditionally used for treating unconsciousness and as “messenger” drugs, regulating and mediating other prescription drugs. Some evidence shows that borneol is a good penetration enhancer, and suggests that borneol acts via a lipophilic fraction that reacts with cell membrane lipid components to mediate the activity of enzymes, carriers, ion channels and receptors [29]–[31]. Muscone can protect against cerebral anoxia and cerebral ischemia, and may enhance the distribution of drugs in brain tissue through opening the BBB [32], [33]. The therapeutic efficacy of traditional Chinese medicine (TCM) often depends on the combined action of a mixture of constituents. In our present study, we conducted geniposide transport studies in the HNEC and evaluated the influence of borneol and muscone on its transport to elucidate the drug-drug interaction mechanisms.

In order to check the influence of aromatic herbs on passive permeability, transport studies were performed using geniposide (50 µg·mL^−1^) as reference compound in the presence of rising concentrations of borneol and muscone. Figure 2 shows the effect of borneol and muscone on the transport profiles of geniposide across the HNEC monolayer. The *P_app_* values of geniposide increased with rising concentrations of borneol and muscone in a dose-dependent manner, and were significantly higher than the control (geniposide 50 µg·mL^−1^).

Borneol is a simple bicyclic monoterpene. When passing through the HNEC monolayer, borneol might loosen the intracellular tight junction and then promote paracellular geniposide transportation. Muscone is a small lipophilic compound and highly lipid-soluble. The permeation enhancement mechanism of muscone might include increasing the permeability of cell membranes or loosening the tight junctions or both. The results indicated that, on the one hand, borneol or muscone could rapidly raise the permeability of cell membrane and promote the absorption of geniposide, but on the other hand, borneol might competitively opening of the tight junction proteins when administrated with muscone simultaneously. So borneol and muscone did not show additive effects on geniposide permeability across HNEC monolayer. The present study showed that the membrane transport of geniposide may occur through the paracellular route, which is widely accepted among pharmaceutical scientists in the membrane transport of hydrophilic drugs [34].

Theoretically, cell morphology and membrane composition determine functionality and drug penetration via the monolayers. The tight junction is envisioned as a zone of dense hydrophobic, intercellular material that would form a seal when two adjacent cells were held close. There are many molecular components of the tight junction (TJ), such as occludins, claudins, junctional adhesion molecules, zonula occludens and actin. TJ proteins define epithelial cell polarity, regulate paracellular permeability and confer barrier function to the epithelia [35]. Among these components, perijunctional actin is known to play a major role in controlling the paracellular permeability, as well as in cell adhesion, polarity, migration and survival [36], [37]. As shown in Figure 3, the outline of stained actin filaments was similar to that reported in the literature [38]. Our results showed that ZO-1 protein was stained in cytoplasm and nucleus (data not shown). As a result, our in vitro studies suggest that borneol and muscone may increase the paracellular permeability of the HNEC monolayer to geniposide due to the opening of the tight junction proteins.

The measurement of the TEER is an easy and quick method to determine the tight junction integrity [39]. In parallel to the permeation experiment, the TEER of the cells was measured. Our results showed that the TEER of the control group did not change during and after the experiment. Further, borneol and muscone using alone or in combination could significantly decrease transepithelial resistance across the in vitro HNEC monolayer (*P*<0.05). The simultaneous presence of borneol and muscone correlates with a larger TEER decrease than those observed in presence of each compound alone. It is quite possible that borneol and muscone may open other tight junctions besides perijunctional actin. However, this process is temporary and reversible. After incubating the cells with BEGM:DMEM/F12 (1∶1) for 24 h, the TEER increased again. A decrease in TEER during the permeation experiment can be explained by the modulation and opening of the tight junctions caused by the permeation enhancer borneol and muscone.

The lipids and proteins in the cell membrane possess certain fluidity. The changes in the cell membrane fluidity could cause many functional alterations of the cells, such as membrane trafficking, drug transport, membrane-bound enzyme activity, cell differentiation and cell recognization [40], [41]. The technique of FRAP has been used to study the lateral mobility of membrane lipids and proteins in a variety of cells and tissues [42]. Results showed that borneol and muscone could significantly increase the cell membrane fluidity of HNEC in a concentration-dependent manner. The order of *R%* in different groups was +Bo (111.2 µg·mL^−1^) Mu (16.68 µg·mL^−1^)>+Bo (111.2 µg·mL^−1^)>+Mu (16.68 µg·mL^−1^)>Ge (50 µg·mL^−1^) (control).

As detected in the previous experiment, borneol can reversibly rearrange the sequence of the phospholipids from the lipid bilayer of the corneal epithelium, make it more regular, and thus, increasing permeation of surface epithelium cells [43]. It is quite possible that borneol facilitates the transmucosa passage of drugs by reducing drag of phospholipids of the lipid bilayer in the nasal epithelium, which is the main barrier for topically applied drugs. The possible mechanism may be that borneol has alcoholic hydroxyl group, which is a hydrogen bond acceptor/donor. Muscone possesses a unique macrocyclic ketone structure, which exhibited high affinity with intercellular lipids. This may cause greater damage to the lipid hydrogen-bond network, and thereby have a greater enhancing effect than using borneol or muscone alone.

The combination effect of opening the tight junction proteins and increasing in membrane fluidity might be suitable to explain the the enhancing effect of borneol and muscone on geniposide transport across the HNEC monolayer here. The hypothesis is supported by a study showing that borneol could obviously loosen the intercellular tight junction in the BBB and accelerate the transportation of substance through the intercellular passage [44]. It could also increase the number and volume of pinocytosis vesicles in BBB cells and then accelerate the transportation of substance by cell pinocytosis [45].

In conclusion, we have demonstrated that the potentiation of nasal uptake of the main active ingredients geniposide in “*Xing Nao Jing*” injection and the enhancing influence of borneol and muscone on its transport through this membrane model. Geniposide was shown to exhibit relatively poor absorption in the HNEC model. It was purely passive diffusion and was not a P-gp substrate. Borneol and muscone behave as effective absorption enhancers in the HNEC monolayer by opening the barrier and increasing the paracellular and transcellular transport. At this stage it is not known how safe the aromatic herbs are for the normal function of HNECs. However, the present study offers an excellent model for further investigation and demonstrates that intranasal administration has potential as an alternative to traditional injection therapy for CNS disorders. More studies on the mechanisms and safety of the application of borneol and muscone as promoters for intranasal brain-targeting delivery are ongoing in our lab.
